# Photonic and Optoelectronic Devices and Systems, 4th Edition

**DOI:** 10.3390/mi17060671

**Published:** 2026-05-29

**Authors:** Muhammad A. Butt

**Affiliations:** Institute of Microelectronics and Optoelectronics, Warsaw University of Technology, Koszykowa 75, 00-662 Warsaw, Poland; ali.butt@pw.edu.pl

The rapid development of photonic and optoelectronic technologies continues to shape progress across integrated photonics, optical sensing, energy harvesting, imaging, communication systems, and semiconductor-enabled optical devices [1,2,3,4]. As modern applications increasingly demand compactness, higher functionality, and improved efficiency, advances in materials, device engineering, and fabrication technologies are steadily transforming the capabilities of optical systems [5,6,7,8]. This 4th edition of the Special Issue Photonic and Optoelectronic Devices and Systems brings together eleven contributions that collectively highlight recent progress across several important areas of photonics and optoelectronics. The Issue includes one review article and ten original research papers covering silicon photonics, metasurfaces, radiation detectors, photovoltaics, photoelectrochemical systems, optical logic, beam shaping, display technologies, LiDAR systems, and wafer level optoelectronic integration. Together, these studies reflect the increasingly interdisciplinary nature of photonics research and demonstrate how innovations in materials, device physics, and system integration continue to drive the field forward [9].

The review article by the guest editor Dr. Butt presents a broad and timely overview of the growing convergence between plasmonics and metasurfaces for next generation planar optical systems [Contribution 1]. The review discusses how the strong field confinement enabled by plasmonic structures can complement the versatile wavefront control offered by metasurfaces within compact and multifunctional optical platforms. Important developments in modulators, detectors, nanolasers, metalenses, beam steering devices, and programmable optical surfaces are critically examined alongside key challenges associated with optical losses, thermal effects, fabrication scalability, and material selection. By bringing together recent developments across plasmonic, metasurface, and hybrid architectures, this work provides a useful perspective on how planar optical systems may continue to evolve for applications in sensing, imaging, communication, and optical information processing.

The ten original research articles in this Issue address a diverse range of topics spanning integrated photonics, photodetection, energy conversion, optical computing, and display technologies. The study by Smolanski and Malka investigates a compact O band 4 × 1 optical power combiner based on a cascaded multimode interference tree configuration implemented on a silicon on insulator platform [Contribution 2]. Designed to operate around 1310 nm, the proposed device employs low loss S bends and adiabatic tapers to achieve efficient coherent power combining while maintaining compactness and fabrication tolerance. Thermal analysis further demonstrates stable operation over a broad temperature range, making the design attractive for O band photonic transmitters and integrated photonic systems requiring efficient power scaling.

In the area of solar energy harvesting, Wang et al. report a novel PN tandem solar cell based on the combination of a dye sensitized TiO_2_ photoanode and a perovskite sensitized NiO photocathode [Contribution 3]. The work addresses an important limitation of tandem dye sensitized solar cells, namely the poor performance of p-type sensitizers, by introducing a hybrid sensitization approach capable of simultaneously improving open circuit voltage and short circuit current density. Through optimization of the photoelectrode thickness and device structure, the authors demonstrate enhanced power conversion efficiency, offering useful insights into future strategies for improving tandem photovoltaic systems.

Infrared polarization sensitive photodetection is explored in the contribution by Zheng et al., who propose a compact chiral mid wave infrared photodetector based on trapezoidal silicon pillars [Contribution 4]. By carefully engineering geometric asymmetry through displacement and tilt angle manipulation, the proposed metasurface enables significantly different responses to left and right circularly polarized light, leading to a high extinction ratio. The compatibility of the design with semiconductor fabrication processes further highlights its practical relevance for integrated infrared sensing, quantum communication, and compact polarization selective photonic systems.

Radiation detection technologies are further advanced in the work by Wang and Li, who propose a novel spiral silicon drift detector featuring a constant cathode gap and arbitrary cathode pitch profiles [Contribution 5]. Unlike conventional concentric ring detector geometries, the proposed design enables improved control of electric field distribution while reducing surface leakage current. Theoretical modeling, together with three dimensional TCAD simulations, reveal smooth electric potential profiles, uniform drift channels, and highly symmetric charge transport. This study provides an important contribution toward the development of high performance detectors for applications in X ray spectroscopy, particle physics, and radiation monitoring.

Freeform optical beam shaping is examined in the contribution by Zhang et al., who present the design and ultra precision fabrication of freeform Fresnel lenses for generating rectangular dark hollow beams [Contribution 6]. By dividing the lens into multiple fan-shaped sectors capable of independently redirecting light toward prescribed spatial locations, the authors successfully demonstrate the generation of square hollow beam profiles. Experimental measurements show strong agreement with simulations, validating the feasibility of the proposed design. The work highlights the practical value of combining freeform optics with Fresnel structures for compact beam shaping applications in optical manipulation, communications, and laser systems.

La Ferrara et al. contribute an interesting study on triple cation perovskite photoanodes for solar water splitting, comparing photovoltaic assisted and immersed photoelectrochemical operating modes [Contribution 7]. By integrating encapsulated mixed halide perovskite solar cells with catalytic nickel foils, the authors demonstrate efficient solar driven water splitting under alkaline conditions while addressing the long standing stability challenges associated with perovskite materials. Particularly noteworthy is the strong performance observed in the immersed configuration, emphasizing the importance of device architecture in maximizing photovoltage utilization and charge transfer efficiency. This work represents a promising step toward scalable and efficient photoelectrochemical systems for hydrogen generation.

All optical information processing in the terahertz regime is explored in the work of Guo et al., who propose dual band all optical logic gates based on unidirectional electromagnetic modes [Contribution 8]. Using a Y-shaped structure capable of supporting robust one way signal propagation, the authors demonstrate several logic operations including AND, OR, NOT, and XNOR functions across different frequency bands. Importantly, the proposed architecture enables simultaneous processing of multiple signals without cross talk, highlighting its potential for future high throughput optical computing and integrated photonic logic systems.

Seo and Park present an energy efficient LiDAR receiver based on a time-to-voltage converter combined with a successive approximation register analog to digital converter [Contribution 9]. Instead of relying on conventional time to digital conversion schemes, the proposed architecture converts time intervals into voltage signals before digitization, significantly reducing circuit complexity and power consumption. Fabricated in a CMOS platform, the receiver demonstrates compactness and low energy consumption while maintaining sufficient temporal resolution for short range LiDAR applications. The proposed approach provides a practical route toward compact sensing systems for autonomous technologies and environmental monitoring.

Display technologies are represented by the contribution of Byun et al., who investigate polydimethylsiloxane-based quantum dot color conversion layers for QD OLED applications [Contribution 10]. By embedding quantum dots within a thermally curable PDMS matrix, the authors successfully suppress blue light leakage while improving overall color conversion efficiency compared with conventional quantum dot only layers. Through systematic optimization of quantum dot concentration and layer thickness, the study demonstrates the benefits of PDMS as a stable and optically transparent host material for future high quality display technologies.

Finally, Zhang et al. report a wafer level transfer process for GaN on Si light-emitting devices based on SiO_2_-to-SiO_2_ direct bonding [Contribution 11]. The proposed strategy combines hybrid planarization with direct bonding to enable efficient transfer of large area wafers while reducing optical losses associated with silicon absorption. Detailed investigations of strain relaxation and optoelectronic performance reveal notable improvements in light output together with important insights into thermal and stress related behavior during substrate transfer. This work offers a practical pathway for advancing high performance GaN based optoelectronic devices and Micro LED technologies.

These eleven contributions collectively provide a timely perspective on several important directions currently shaping photonic and optoelectronic research. A noticeable theme across the Issue is the increasing emphasis on compact and multifunctional optical systems, whether through integrated silicon photonics, metasurface-enabled photodetection, freeform beam shaping, wafer level integration, or energy efficient sensing and computation. At the same time, advances in renewable energy devices, radiation detection, and display technologies illustrate how innovations in materials and device engineering continue to expand the practical reach of photonic technologies. This Special Issue highlights not only the diversity of modern photonics research but also the growing convergence between optical physics, semiconductor technologies, materials engineering, and system level design. We hope that the studies presented here will stimulate further developments and encourage continued efforts toward compact, efficient, and application driven photonic and optoelectronic systems.
